# Detection of plasmid-mediated tigecycline-resistant gene *tet*(X4) in *Escherichia coli* from pork, Sichuan and Shandong Provinces, China, February 2019

**DOI:** 10.2807/1560-7917.ES.2019.24.25.1900340

**Published:** 2019-06-20

**Authors:** Li Bai, Pengcheng Du, Yinju Du, Honghu Sun, Pei Zhang, Yuping Wan, Qi Lin, Séamus Fanning, Shenghui Cui, Yongning Wu

**Affiliations:** 1 Key Laboratory of Food Safety Risk Assessment, National Health Commission of the People’s Republic of China, Beijing, People’s Republic of China; 2 Food Safety Research Unit of Chinese Academy of Medical Sciences, China National Center for Food Safety Risk Assessment, Beijing, People’s Republic of China; 3 These authors contributed equally to this work; 4 Institute of Infectious Diseases, Beijing Ditan Hospital, Capital Medical University, and Beijing Key Laboratory of Emerging Infectious Diseases, Beijing, People’s Republic of China; 5 Center for disease control and prevention of Liaocheng city, Liaocheng, People’s Republic of China; 6 Chengdu institute for Food and Drug Control, Chengdu, People’s Republic of China; 7 UCD-Centre for Food Safety, School of Public Health, Physiotherapy and Sports Science, University College Dublin, Belfield, Dublin, Ireland; 8 Department of Food Science, National Institutes for Food and Drug Control, Beijing, People’s Republic of China

**Keywords:** *Escherichia coli*, conjugative plasmid, tigecycline resistance

## Abstract

The plasmid-mediated high-level tigecycline resistance gene, *tet*(X4), was detected in seven *Escherichia coli* isolates from pork in two Chinese provinces. Two isolates belonged to the epidemic spreading sequence type ST101. *Tet*(X4) was adjacent to IS*Vsa3* and concurrent with *floR* in all seven isolates. In addition to IncFIB, the replicon IncFII was found to be linked to *tet*(X4). This report follows a recent detection of *tet*(X3)/(X4) in *E. coli* from animals and humans in China.

It has been speculated that one in five resistant human infections are caused by antibiotic resistant bacteria originating from food and animals [1]. China is the world's biggest producer and consumer of pork and has trade links with many countries [2]. In May 2019, He et al. reported the finding of two transferable plasmid-mediated tigecycline resistance genes, *tet*(X3) and *tet*(X4) [3]. These genes were detected in numerous Enterobacteriaceae and *Acinetobacter* from animals and meat for consumption (chicken and pork) in three representative provinces of China located in different geographical areas, as well as from patients originating from 20 hospitals in 20 different cities of the country [3]. Both genes conferred clinically-significant levels of tigecycline resistance (minimum inhibitory concentration, MIC ≥ 32 mg/L) [3]; the tigecycline breakpoints for Enterobacteriaceae and *A. baumannii* are MIC > 0.5 mg/L according to the European Committee on Antimicrobial Susceptibility Testing (EUCAST) [4]. The newly emerging, rapid and widespread dissemination of *tet*(X3) and *tet*(X4) illustrated a flux mediated by horizontal gene transfer representing a paradigm shift in tigecycline resistance, which until now had only been found to be spread by vertical transmission mechanisms [3]. This currently poses a further threat to public health, as the emergence of these transferable tigecycline resistance genes in food-producing animals could potentially lead to an increased risk of infection by strains harbouring these genes and treatment failure in humans [3].

## *Escherichia coli* harbouring transferable *tet*(X4) obtained in this study

**Figure 1:**
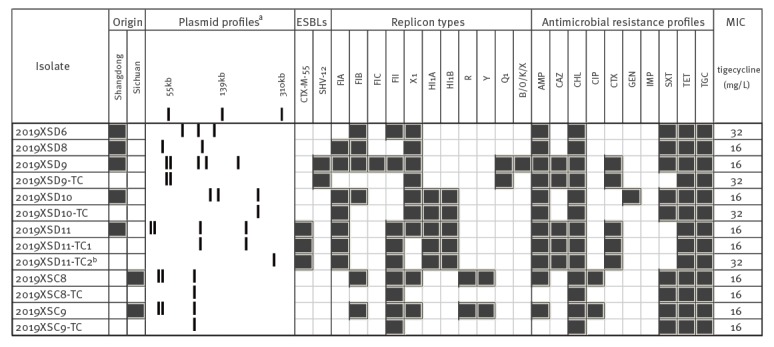
A heat-map showing the comparison of the *Escherichia coli* donors and the resultant transconjugants, characterised on the basis of their origins, plasmid profiles; ESBL-types; replicon types; susceptibility profiles and MIC of tigecycline, China, February 2019

AMP: ampicillin; CAZ: ceftazidime; CHL: chloramphenicol; CIP: ciprofloxacin; CTX: cefotaxime; ESBL: extended spectrum beta-lactamase; GEN: gentamicin; IMP: imipenem; MIC: minimum inhibitory concentration; SXT: trimethoprim/sulfamethoxazole; TET: tetracycline; TGC: tigecycline.

In this study, we sought bacteria harbouring the newly reported tigecycline resistance genes in two provinces in China using the method reported by He’s study [3]. Seven *tet*(X4) positive isolates (20.6%, 95% confidence interval (CI): 8.7–37.9), were recovered from 34 retail pork samples taken in Sichuan (8.7%, 2/23, 95%CI: 1.1–28.0) and Shandong (45.5%, 5/11, 95%CI: 16.7–76.6) Provinces in February, 2019. The *tet*(X4) sequences in all seven isolates were identical to that reported by He et al. [3]. No *tet*(X3) was detected. All isolates were identified as *Escherichia coli* by VITEK 2 and 16S rDNA-based sequencing. The MICs against tigecycline ranged from 16 to 32 mg/L, with all isolates expressing resistance to the majority of antimicrobial agents tested for in this study except meropenem (Table). All isolates were multidrug-resistant (MDR), in that they were resistant to three or more different classes of antimicrobials and two were confirmed as extended spectrum beta-lactamase (ESBL)-producing (denoted as 2019XSD9 and 2019XSD11). S1-pulsed-field gel electrophoresis (PFGE) profiling showed that all seven isolates possessed multiple plasmids of differing sizes (Figure 1). Of the seven isolates, five successfully transferred the *tet*(X4)-mediated resistance phenotype via conjugation to *E. coli* J53 with transfer frequencies from 3.5 × 10 ^− 6^ to 2.7 × 10 ^− 1^ being recorded. The *tet*(X4) gene in transconjugants was confirmed by PCR. Each of the transconjugants had one or two plasmids ranging in size from ca 50- to 280-kbp (Figure 1). The MICs against tigecycline were re-assessed in these cases and a > 64-fold increase was recorded, compared with the plasmid-less recipient (*E. coli* J53, 0.125 mg/L).

**Table:** Characteristics of *tet*(X4) positive *Escherichia coli* isolated from pork samples and their serotypes, sequence types, antimicrobial resistance profiles and resistance determinants along with their minimum inhibitory concentrations to tigecycline, Sichuan and Shandong Provinces, China, February 2019

Isolates	Serotypes	Sequence types	Antimicrobial resistance profiles	Tigecycline MIC (mg/L)	Additional resistance determinants identified by WGS
2019XSD6	O82:H8	ST101	AMP-CHL-SXT-TET-TGC	32	*qnrS1, aadA2, bla*_TEM-1C_, *dfrA14, floR, lnu*(F), *tet*(X4)
2019XSD8	O5:H32	ST761	AMP-CHL-SXT-TET-TGC	16	*qnrS1, bla*_TEM-1B_, *dfrA5, floR, mef*(B), *sul3, tet*(A), *tet*(M), *tet*(X4)
2019XSD9	O109:H40	ST101	AMP-CAZ-CHL-CTX-SXT-TET-TGC	16	*aadA2, bla*_SHV-12_, *floR, lnu*(F), *strA, strB, sul2, bla*_TEM-1B_, *tet*(X4)
2019XSD10	O126:H2	ST10	AMP-CHL-GEN-SXT-TET-TGC	16	*qnrS1, aac(3)-IId, aadA2, aadA22, bla*_TEM-1B_, *dfrA12, erm*(42), *floR, mef*(B), *mph*(A), *sul3, tet*(A), *tet*(X4)
2019XSD11	ONT:H25	ST847_similar	AMP-CAZ-CHL-CTX-TET-TGC	16	*qnrS1, aadA22, bla*_CTX-M-55_, *bla*_TEM-1B_, *floR, fosA, strA, strB, sul2, tet*(A), *tet*(X4)
2019XSC8	O5:H11	ST48	AMP-CHL-CIP-SXT-TET-TGC	16	*qnrS2, aph(3')-Ia, bla*_TEM-1B_, *floR, sul2, sul3, tet*(A), *tet*(M), *tet*(X4)
2019XSC9	O5:H11	ST48	AMP-CHL-CIP-SXT-TET-TGC	16	*qnrS2, aph(3')-Ia, bla*_TEM-1B_, *floR, sul2, sul3, tet*(A), *tet*(M), *tet*(X4)

AMP: ampicillin; CAZ: ceftazidime; CHL: chloramphenicol; CIP: ciprofloxacin; CTX: cefotaxime; GEN: gentamicin; MIC: minimum inhibitory concentration; SXT: trimethoprim/sulfamethoxazole; TET: tetracycline; TGC: tigecycline; WGS: whole genome sequencing.

The MIC for tigecycline in each group combination is also shown. In the first column, the letters ‘TC’ typify the transconjugants. Black and white squares denote the presence and absence, respectively of a particular feature.

^a^ The size of the plasmids was determined according to the S1-pulsed-field gel electrophoresis results.

^b^ The plasmid profile of 2019XSD11-TC2 is due to a fusion in the transconjugant of two plasmids originating from the donor strain (data not shown).

## Multiple subtypes harbouring highly variable plasmids carrying *tet*(X4)

The genomic characteristics of these *tet*(X4) positive isolates were further investigated. Firstly, PFGE exhibited genetically divergent pulsotypes with identities from 55.8% to 85.0% (Supplementary Figure S1). Whole genome sequencing (WGS) was performed and the Illumina reads were submitted to the National Center for Biotechnology Information (NCBI) Sequence Read Archive (SRP192184). In silico multilocus sequence typing (MLST) analysis of WGS data identified five known sequence types (ST) along with one new type as described in the Table. Two of the isolates (2019XSD6 and 2019XSD9) belonged to ST101. Another two isolates (2019XSC8 and 2019XSC9) belonged to ST48 and expressed the same antibiotic resistance phenotype, having an indistinguishable S1-PFGE profile (Figure 1). However, by mapping the sequencing reads to an earlier *E. coli* WCHEC1613 (ST48) cultured from the sewage of a hospital [5], we identified 20 high quality single nt polymorphisms (hqSNPs) between 2019XSC8 and 2019XSC9, indicating that the two isolates were not identical. Several antibiotic resistance genes along with *tet*(X4) were identified, including but not limited to *bla*_CTX-M-55_, *qnrS1, qnrS2, floR, fosA* and *mph*(A) (Table). These results were in line with the MDR phenotype.

## Genetic characterisation of plasmids harbouring the *tet*(X4) gene

**Figure 2:**
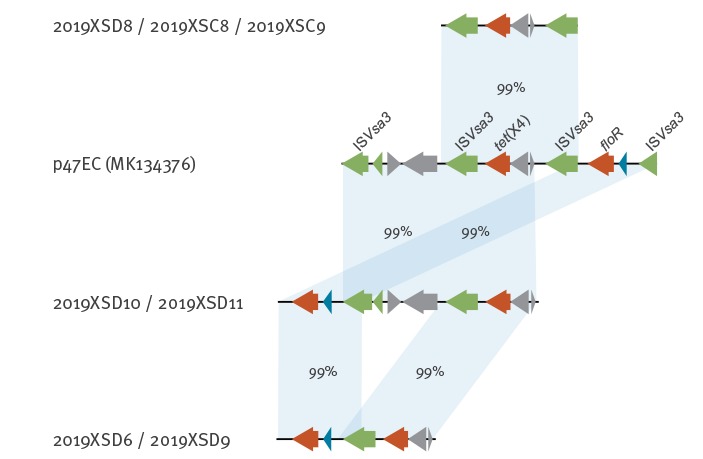
Gene alignments of the *tet*(X4) transposon in isolates from this study with that of p47EC reported by He et al., China, February, 2019

We subsequently analysed the detailed genetic context of the *tet*(X4) harbouring plasmids along with the other resistant determinants. In the study reported by He et al., the *tet*(X4) harbouring plasmid p47EC from *E. coli* was 170 kbp, and the *tet*(X4) gene was carried within a cassette flanked by IS*Vsa3* [3]. In the current study, both *tet*(X4) and *floR* (encoding resistance to phenicols) were identified in all seven isolates, and the gene cassette comprised by *tet*(X4) and IS*Vsa3* was conserved at > 99% identity with that in plasmid p47EC (Figure 2).

The arrows in different colours represent genes of differing functions (red: antibiotic resistance gene; blue: transfer associated gene; green: integrase, recombinase or transposase; grey: gene of other function). The light grey blocks represent the alignment region between two sequences and the identities are marked on the blocks. The p47EC was reported by He et al. [3].

Plasmids harbouring *tet*(X4) have been reported to be related to IncFIB replicon types in *E. coli*. In this study, identification of types IncFII, IncFIA, IncHIA and IncHIB now appear to expand this repertoire of *tet*(X4)-associated plasmids (Figure 1).

## Discussion

In this study, we detected tigecycline resistant *E. coli* (20.6%), positive for *tet*(X4), from pork in two provinces in China 2019. Most *tet*(X3) or *tet*(X4)-positive strains reported by He et al. also originated from pigs, and among bacteria positive for such genes, *E. coli* was the predominant species [3]. Taken together, the results suggest that *E. coli* strains positive for *tet*(X3) and *tet*(X4) might exhibit a broad geographical distribution having already spread in some areas of China. Interestingly, *tet*(X4) was located on various conjugative plasmids of diverse replicon types. These observations suggested that *tet*(X4) could be captured by a range of mobile genetic elements circulating among bacterial strains (Figure 1), a scenario reminiscent of *mcr-1* [6].

In silico MLST analysis enabled us to identify two isolates belonging to ST101. This ST was reported earlier to be common among ESBL-producing *E. coli* recovered from meat products imported into the European Union (EU) from non-EU countries [7], a finding potentially suggesting that the international trade of food products, which is expanding, could present a route for dissemination of antibiotic-resistant food-borne pathogens. ST101 has been reported previously in 15 countries, and is also frequently associated with NDM-1 [8,9]. In this context, and given the finding of *tet*(X4)-positive ST101 *E. coli* in the current study, it is interesting to speculate that ST101 might represent a convenient and efficient way of spread for the *tet*(X4)-mediated resistance mechanism,**thereby posing a serious challenge to public health.

The other two* tet*(X4)-positive isolates reported here (2019XSC8 and 2019XSC9) belonged to ST48. These were cultured from two samples obtained at the same retail outlet on the same day in Sichuan province and had 20 hqSNPs difference. Given this difference, it is unlikely that the isolates originated from cross-contamination of meat products at the outlet. This could rather point to a possible origin by clone-like transmission either within a given live animal (if samples originated from the same pig) or among different pigs following cross-colonisation. Under the scenario of transmission within one single animal, the SNP-based genomic differences identified would suggest that intra-host variation [10] was stable. Colonisation/adaptation in the animal host, however, remains to be confirmed.

Tigecycline is not licensed for veterinary use. However, the production and use of tetracyclines are highest among all of the antimicrobial compounds in China [11,12]. It has been predicted that Tet(X) might become the most problematic future Tet determinant given its weak intrinsic tigecycline-resistance activity [13] concomitant with the historical selective pressure exerted by large scale use of earlier generations of this class of antibiotics.

Our findings demonstrate that high-level tigecycline resistant *E. coli* from pork harbouring the *tet*(X4) gene were associated with promiscuous plasmid types, resulting in a diverse range of clones. With international trade of food-producing animals and products derived from them, along with travel, *tet*(X4)-positive tigecycline resistant *E. coli* could represent the emergence of an additional antimicrobial mechanism of concern. Understanding the transmission mode of these genes allied to active surveillance of plasmid-mediated *tet*(X3)/(X4) variants in bacteria based on a one health approach is urgently recommended to support improvements in infection control practices to limit its further dissemination.

## Supplementary Data

235000 Supplementary Figure S1
