# Supplementary material for: Detection of plasmid-mediated tigecycline-resistant gene tet(X4) in Escherichia coli from pork, Sichuan and Shandong Provinces, China, February 2019
Source: Euro Surveill. 2019 Jun 20;24(25):1900340. doi: 10.2807/1560-7917.ES.2019.24.25.1900340 (PMC6593906; doi:10.2807/1560-7917.ES.2019.24.25.1900340)

This supplementary material is hosted by *Eurosurveillance* as supporting information alongside the article ‘Detection of plasmid-mediated tigecycline-resistant gene *tet*(X4) in *Escherichia coli* from pork, China, February 2019’ on behalf of the authors who remain responsible for the accuracy and appropriateness of the content. The same standards for ethics, copyright, attributions and permissions as for the article apply. *Eurosurveillance* is not responsible for the maintenance of any links or email addresses provided therein.

Supplementary Figure S1. PFGE pattern for seven *tet*(X4) positive tigecycline-resistant isolates.

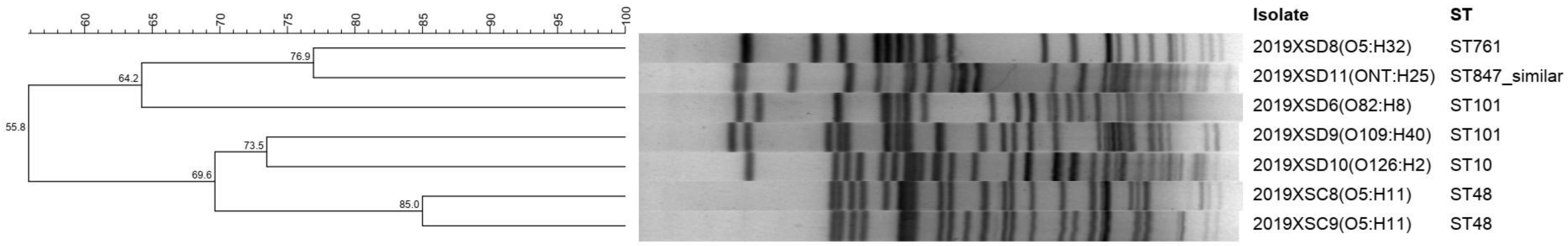

Supplement: Supplementary Figure S1 [file 1900340_WU_SupFigS1.pdf]
